# OR13-005 – Investigation of clinical and laboratory significance of TNFRSF1A intron by reverse-phase protein microarray

**DOI:** 10.1186/1546-0096-11-S1-A267

**Published:** 2013-11-08

**Authors:** E Drewe, O Negm, W Abduljabbar, P Hawkins, L Fairclough, I Todd, P Tighe

**Affiliations:** 1 Clinical Immunology and Allergy, Nottingham University Hospitals NHS Trust, UK; 2 Molecular Medical Science, University of Nottingham, Nottingham, UK; 3 National Amyloidosis Centre, London, UK

## Introduction

Genetic analysis for autoinflammatory disease may reveal sequence changes of uncertain clinical significance. We describe investigation of a 26 year old with unexplained inflammatory symptoms with a novel intronic change in TNFRSF1A (intron 4 c.472+88 C>A) by examining reverse-phase protein microarray. In addition we describe clinical symptoms and response to anakinra and etanercept.

## Objectives

To assess whether this subject with intronic change had laboratory and clinical features which would confirm a diagnosis of TRAPS.

## Methods

Clinical history was documented along with clinical response to prednisolone, anakinra and etanercept. Intracellular signaling pathways that govern inflammation associated with TNFRSF1A signaling pathways were examined in peripheral blood mononuclear cells (PBMC) using reverse-phase protein microarray. Profiles were compared between the subject with intronic change, C33Y TRAPS subjects and matched healthy control.

## Results

Clinical history confirmed some clinical features of TRAPS with fevers up to 40 degrees C from childhood and frequent episodes of myalgia, vomiting and abdominal pain lasting up to 4 weeks. On many occasions, however, symptoms were associated with normal CRP and SAA. Symptoms responded to oral prednisolone but no benefit occurred with anakinra. Subsequent treatment with etanercept resulted in less frequent and severe attacks.

Reverse phase protein microarray technology of signaling pathways has previously demonstrated that PBMC from patients with C33Y TRAPS show subtle upregulation of NF-kB, p38, MEK/ERK and JNK MAP kinase pathways, Phosphinositide 3 kinase, STAT3, JAK2/c-Src, GSK-3beta and transcription factors including ATF, Elk and Jun. The microarray from subject with intronic change did not show any significant variations compared to healthy control and did not resemble a C33Y TRAPS profile. However some proteins such as pERK1/2, pP38 and TRAF6 were downregulated in subject intron compared to control.

## Conclusion

This subject with intronic change lacks some typical clinical features of TRAPS such as lack of acute phase response and responsiveness to anakinra. In addition protein array profile did not resemble that of C33Y TRAPS patient. This subject does however have a chronic recurrent febrile illness associated with TNFRSF1A intronic change and improvement on etanercept, suggesting other mechanisms could be implicated.

## Disclosure of interest

None declared

